# A Web-Based Training Program for School Staff to Respond to Self-Harm: Design and Development of the Supportive Response to Self-Harm Program

**DOI:** 10.2196/50024

**Published:** 2024-06-04

**Authors:** Anne-Marie Burn, Poppy Hall, Joanna Anderson

**Affiliations:** 1 Department of Psychiatry University of Cambridge Cambridge United Kingdom

**Keywords:** self-harm, schools, young people, youth, school staff, training, coproduction, qualitative

## Abstract

**Background:**

Self-harm is common among adolescents and is a major public health concern. School staff may be the first adults to notice a young person’s self-harm and are well placed to provide support or signpost students to help. However, school staff often report that they do not feel equipped or confident to support students. Despite the need, there is a lack of evidence-based training about self-harm for school staff. A web-based training program would provide schools with a flexible and cost-effective method of increasing staff knowledge, skills, and confidence in how to respond to students who self-harm.

**Objective:**

The main objective of this study was to coproduce an evidence-based training program for school staff to improve their skills and confidence in responding to students who self-harm (Supportive Response to Self-Harm [SORTS]). This paper describes the design and development process of an initial prototype coproduced with stakeholders to ensure that the intervention meets their requirements.

**Methods:**

Using a user-centered design and person-based approach, the SORTS prototype was informed by (1) a review of research literature, existing guidelines, and policies; (2) coproduction discussions with the technical provider and subject matter experts (mental health, education, and self-harm); (3) findings from focus groups with young people; and (4) coproduction workshops with school staff. Thematic analysis using the framework method was applied.

**Results:**

Coproduction sessions with experts and the technical provider enabled us to produce a draft of the training content, a wireframe, and example high-fidelity user interface designs. Analysis of focus groups and workshops generated four key themes: (1) need for a training program; (2) acceptability, practicality, and implementation; (3) design, content, and navigation; and (4) adaptations and improvements. The findings showed that there is a clear need for a web-based training program about self-harm in schools, and the proposed program content and design were useful, practical, and acceptable. Consultations with stakeholders informed the iterative development of the prototype.

**Conclusions:**

SORTS is a web-based training program for school staff to appropriately respond to students who self-harm that is based on research evidence and developed in collaboration with stakeholders. The SORTS program will equip school staff with the skills and strategies to respond in a supportive way to students who self-harm and encourage schools to adopt a whole-school approach to self-harm. Further research is needed to complete the intervention development based on the feedback from this study and evaluate the program’s effectiveness. If found to be effective, the SORTS program could be implemented in schools and other youth organizations.

## Introduction

### Self-Harm and Young People

Self-harm (self-injury and self-poisoning) is a major global public health concern [1]. The rates of self-harm among young people, particularly those in their mid to late teenage years, has steeply risen [2], with reported prevalence in the United Kingdom at nearly 20% [1]. Early reports suggest that the COVID-19 pandemic is likely to have a long-term, negative effect on young people’s mental health [3], potentially increasing rates of suicide and self-harm [4]. Young people who self-harm are at high risk of adverse outcomes, including poor mental health in adulthood and an elevated risk of suicide [1,5]. Research indicates that approximately 19% of those who self-harm in adolescence are still doing so 10 years later, suggesting that it can be a persistent behavior [6]. Most young people who self-harm do not seek any formal or informal support as they are often unsure of who to speak to, try to cope with their problems alone, or believe that no one can help [7,8].

### The Role of Schools

Schools play an essential role in early detection of students’ mental health difficulties [9] and supporting young people’s mental health [10]. Indeed, school staff are often the first professionals to notice a young person’s self-harm and are well placed to identify early signs and intervene to prevent severe outcomes [11]. However, many staff members report that they do not feel equipped or confident to respond to students who are self-harming and are fearful of responding in the wrong way [12-14]. This can result in negative or dismissive reactions to discovery or disclosure of self-harm, which in turn can increase a young person’s motivation to keep their behavior secret and decreases the likelihood of seeking help [7]. Some schools perceive disclosures of self-harm as a safeguarding concern, and their default response is to inform parents, which, if self-harm is a result of family difficulties, can aggravate rather than alleviate the problem. At worst, this can prevent young people from disclosing at school [15] and limit opportunities for adults to intervene.

### Need for School Training on Self-Harm

Research shows that there is a need for staff training and policies to help schools address self-harm [16-20]. Currently, there are no national-level UK policies outlining how schools should respond to self-harm, and it is up to each school to develop and implement their own policies. There are a few information resources available for schools about self-harm and how to respond [21-23], but the evidence base for these resources is currently lacking.

A recent systematic review looked at the effectiveness, feasibility, and acceptability of training programs and support tools for school staff to respond adequately to young people who disclose self-harm [18]. The review found only 8 studies, which comprised 4 training programs, 2 workshops, a school policy, and a website, and all were based in Australia or the United States. The quality of the included studies ranged from weak to moderate, and there was an absence of randomized controlled trials and scarcity of follow-up data. The poor description of training content, lack of feasibility outcomes, and overall low quality of the included studies limit the suitability of these training programs for implementation in UK schools. Although participation in a training program increased staff confidence and knowledge regarding how to respond to pupils who self-harm, most programs lacked follow-up or refresher sessions. There was a significant decline in school staff’s perceived and objective knowledge over time, and participants expressed the need for additional ongoing support. The current best practice in mental health training stresses the importance of periodic follow-up to ensure appropriate implementation of skills learned [23]. These findings highlight a clear need for staff training and policies to help school staff address student self-harm and the importance of having ongoing access to training to help participants consolidate and sustain acquired knowledge and skills.

In a UK survey, just over half of school staff reported having received some training on self-harm, but only 22% rated the training as good [21]. Staff wanted greater organizational support, knowledge, and skills to effectively support pupils who self-harm [13]. School staff reported that a lack of time, training, and resources are barriers to effectively addressing self-harm and they would like good-quality evidence-based training [18]. Web-based training programs can be developed and maintained at a relatively low cost [24]. The cost of delivery is also very low compared to programs delivered face-to-face [25], and there is evidence suggesting that web-based teacher training is more cost-effective compared to training delivered in person [26,27]. Web-based programs, unlike those delivered face-to-face, are flexible and can be completed at a pace and time convenient for users, which is particularly important for busy school staff [25,28,29]. There is evidence suggesting that low cost and flexibility increase the feasibility and acceptability of school-based programs and contribute to their successful implementation and adoption [30].

### This Study

There is a paucity of evidence-based programs designed specifically for school staff [13]. To address this need, this study aimed to coproduce a web-based training resource for school staff to respond to students who self-harm that could be implemented in schools. This early prototype development involved collaborative work with different stakeholders, including young people, school staff, and experts in mental health and self-harm. Coproduction ensures that the knowledge generated is shaped by the requirements of the target users and remains relevant within their specific local context [31]. Involving stakeholders at all stages of intervention development aligns with the Medical Research Council guidelines for complex interventions [32], and this approach is particularly important for interventions targeting complex social systems [33]. In this paper, we describe the design and development of the Supportive Response to Self-Harm (SORTS) prototype, which serves as an exemplar of how involvement of stakeholders can inform the development of web-based training programs and optimize uptake and user engagement.

## Methods

### Ethical Considerations

Ethics approval was granted by the University of Cambridge Department of Psychology Ethics Committee (reference: PRE.2022.053). Electronic consent was obtained from participants, and parental consent was obtained for young people aged <16 years. Participants were informed that their data would be kept confidential and that no individuals would be personally identifiable in the data collected in the study. Transcripts were deidentified and each participant was given a unique alphanumeric code. All participants completed a web-based demographic form before the sessions. Young people received a £20 (US $24.74) shopping gift voucher and school staff received a £40 (US $49.48) gift voucher as a thank you for their time.

### Public Involvement in the Study Design

Considering the sensitivity of the topic in this research, it was important to engage with members of the public from the start of the study to ensure that the study aims, procedures, and public-facing documents were acceptable and accessible. We held meetings with 9 members of the public (young people: n=4, 44%; parents: n=1, 11%; school staff members: n=2, 22%; and members of a young people’s mental health charity: n=2, 22%) to advise on study procedures and steer the project. Members reviewed the study materials (eg, participant information sheets and consent forms) and gave feedback on the proposed methods and topic guides. Early feedback from members of the public helped shape the study design and considerably improved the study materials, ensuring that public-facing documents were easy to read for potential participants.

### Logic Model

The theoretical underpinning of the SORTS program is the self-efficacy theory by Bandura and Adams [34]. Bandura and Adams [34] describe self-efficacy as one’s beliefs about their capabilities to plan and execute actions that are required to produce a desired outcome. Studies show that teachers with a high level of self-efficacy are more confident and effective in their teaching as well as in classroom management and are viewed by students as more competent and trustworthy [35]. Teachers’ attitudes and confidence in supporting students’ mental health may be determined by their knowledge, interests, past experience, and self-efficacy [36]. The theory of change hypothesis is that completing the SORTS training program will lead to improved school staff knowledge about self-harm and increased confidence when responding to students who self-harm, which in turn will facilitate student help seeking and earlier access to support. The theory of change will be revised iteratively throughout the project as new findings emerge.

The SORTS training program aims to (1) improve staff knowledge of self-harm, (2) build staff confidence when responding to self-harm, (3) provide resources for schools to support students, and (4) foster a whole-school approach to self-harm. We developed a logic model (Multimedia Appendix 1) based on the literature and relevant theory. This shows how the SORTS training program might lead to an improvement in school staff’s knowledge about self-harm, increase their confidence when responding to students who self-harm, and facilitate student help seeking and early access to support. Moreover, it shows the mechanisms through which the intervention would be expected to work to support staff and improve outcomes for young people and provides a theoretical framework that will be tested in the next evaluation phase.

### Research Plan

This study was underpinned by a user-centered design and person-based approach, which uses in-depth qualitative methods to capture the perspectives of potential end users and other relevant stakeholders throughout the stages of intervention development and evaluation [37-39]. Gaining insight into people’s views at the early stage of intervention planning, combined with existing theory and evidence, can inform the aims and key characteristics of the planned intervention and increases intervention acceptability and feasibility [39-41].

**Figure 1:**
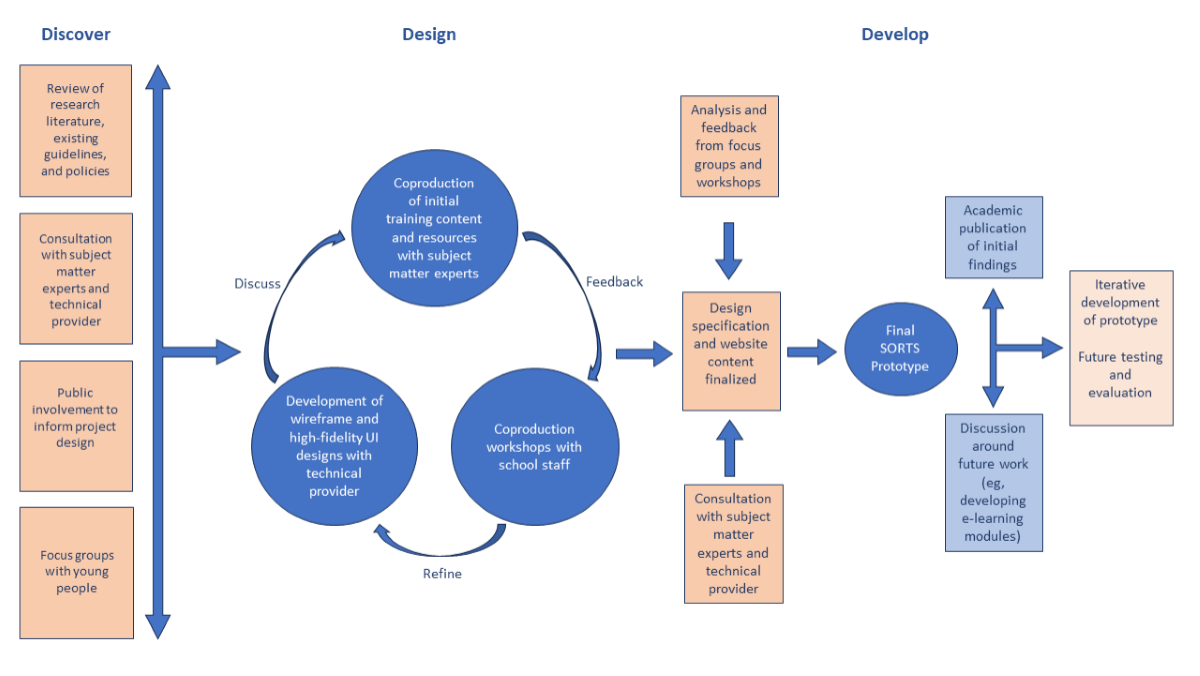
Framework for coproduction and prototype development. SORTS: Supportive Response to Self-Harm; UI: user interface.

We commissioned a technical developer at the start of the project to create a high-fidelity user interface (UI) design and concepts and held a series of coproduction meetings with a panel of subject matter experts to develop the training content. In parallel, we conducted focus groups with young people to understand their expectations of how schools can best support pupils who self-harm. Focus groups are a particularly useful method of data collection in exploratory research because they allow the conversation to move in directions that may not have been anticipated by the researchers [39]. Findings from focus groups with young people were carried forward to the co-design workshops, where we discussed staff’s views and knowledge of self-harm and shared the training content and UI designs. Consultations with staff informed the final prototype specification. Figure 1 shows how the prototype was informed by the literature, focus groups, workshops, and other consultations.

### Procedures

#### Coproduction of Initial Training Content and Designs

We convened a panel of 4 mental health and self-harm experts experienced in working with young people in educational settings and invited them to work with us to develop the training content and UI designs. Expert advisors included 2 senior school leaders responsible for mental health support provision in their schools and for training staff in mental health topics. The panel also included 2 representatives from the Charlie Waller Trust, a leading mental health charity that delivers training about self-harm in schools, colleges, and universities in the United Kingdom; 1 representative is an accredited counselor and psychotherapist, and the other has 20 years’ experience of working in the mental health field and delivering training to school staff about self-harm. They were involved in a coresearcher capacity, whereby they collaborated with the research team to advise on knowledge areas, skills, and techniques that should be incorporated in the training as well as guidance on plans for implementation and dissemination. Meetings with expert advisors began at the start of the study and continued throughout intervention development to ensure that the content and delivery would be relevant, accessible, and acceptable.

Drawing on the findings from the literature, including the team’s recent systematic review [13] and existing guidelines and policies [42], we coproduced a draft of the training content with the expert panel. It incorporated evidence-based information about self-harm, including how to spot signs, appropriate responses to disclosure, and strategies to address recurrent episodes. Some of the proposed tools were knowledge quizzes, video clips, sound clips, techniques to practice different responses, and communication skills. The aim was to produce enough content to sufficiently cover the topic at a level that would provide a basic training for all school staff rather than being aimed at staff who already have advanced knowledge of mental health. A resource toolkit was also developed in collaboration with the expert panel to help staff support pupils or to direct young people to appropriate help.

In addition, the technical provider developed high-fidelity mock-ups of the UI design and a wireframe, which would enable us to communicate the UI design and underlying core functionality to school staff and stimulate feedback in the coproduction workshops. The wireframe showed how the content would be organized and navigated and gave examples of techniques for users to practice and consolidate new skills. Using a wireframe and visual mock-ups of the UI at this early stage was an efficient way to gain feedback from potential users before embarking on expensive and time-consuming prototype building. We held regular meetings with the technical developer to provide feedback from the expert advisors and stakeholders to inform the ongoing prototype development.

#### Recruitment for Focus Groups and Workshops

We recruited young people aged 14 to 21 years who had a history of self-harm or knew someone who self-harmed. In addition, we recruited secondary school staff working in a range of teaching and nonteaching roles, including teachers, student support staff, school mental health leads, and members of the senior leadership team (SLT; eg, assistant head teachers).

The recruitment strategy involved contacting schools in the East of England to ask whether they would participate by raising awareness of the study with their students and staff. Schools were based in areas of deprivation and high mental health need [43] as well as more affluent areas. A key contact at the schools distributed the study flyer and displayed posters, which provided information about the study and the contact details of the study team (Multimedia Appendix 2). To reach school leavers in the 18-21 years age group, details of the study were posted on social media (eg, Facebook) and through the research team’s existing networks, followed by snowball recruitment.

Participants who expressed an interest were provided with a study information sheet including the aims of the research and details about consent, confidentiality, and data protection. The information sheets explicitly stated that participants would not be asked to discuss their own or others’ self-harm behavior.

#### Focus Groups With Young People (Discover)

A semistructured topic guide was developed for the focus groups with young people (Multimedia Appendix 3). We held 2 focus groups with a total of 11 young people to understand their general views on how schools discuss and manage self-harm and their expectations of how schools should respond to self-harm and generate ideas to inform the prototype development. We presented vignettes at the start of the sessions to provide example scenarios of school staff responding to student self-harm. Vignettes are a particularly useful method for discussing sensitive topics and mean that participants do not feel they have to speak from personal experience [44]. Focus groups explored young people’s experiences of school staff addressing self-harm (ie, where school staff responded well, poorly, or not at all) and issues regarding disclosure, including the chosen time or particular person to disclose to. In addition, we asked about their views on what staff should know and understand about self-harm. Before the focus groups, the researchers were able to brief young people about the topics that would be covered in the session to allay any concerns they might have. Due to the sensitivity of the topic, the focus group numbers were limited to 6 participants as this would allow everyone time to speak in the time allotted. Focus groups were facilitated by 2 members of the team (AMB and PH) who are both experienced in working with young people and discussing mental health issues. One focus group was conducted on the web for practical reasons because young people were recruited over a wide geographical area, and another session was held in person with students at a school in the East of England.

#### Coproduction Workshops With Staff (Design)

We held 3 co-design workshops with school staff at 2 schools in the East of England. The aim was to collect feedback from school staff regarding the proposed training content and delivery. This included assessing the perceived comprehensiveness of the training, its effectiveness in developing and reinforcing new knowledge and skills, and whether it encouraged user engagement (Multimedia Appendix 4).

At 2 weeks before the workshops, school staff were asked to complete a pretask that involved reading a subsection of the training content and completing an evaluation feedback form that would be discussed at the workshops. This was to give participants ample time to read and digest their allocated section in preparation for the workshop discussions. Because of the number of participants, the entire training program was thoroughly reviewed.

Workshops were conducted in person on school premises, lasted approximately 2 hours, and were facilitated by 2 members of the research team (AMB and PH) and a member of the technical development team. The session began with the lead facilitator giving a presentation about the objectives of the study and the plan for the workshops. Discussions began by asking school staff to share their thoughts about self-harm in schools and about any training they may have had.

**Figure 2:**
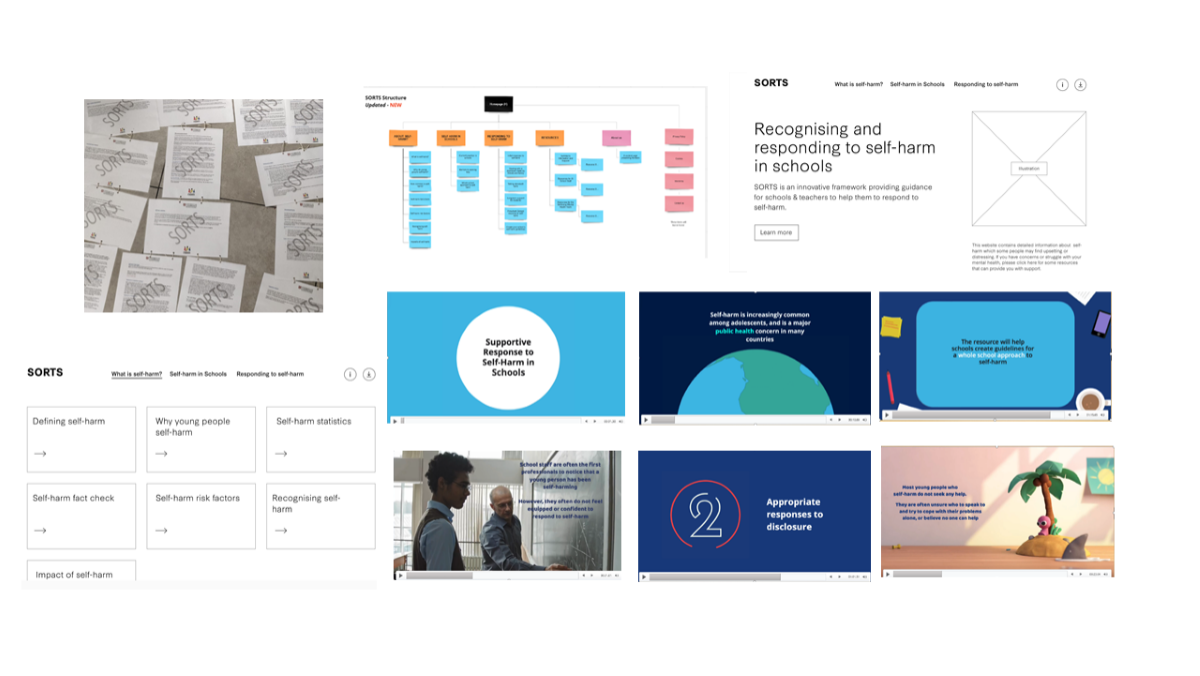
Low-fidelity mock-up of the training content, wireframe, and video examples. SORTS: Supportive Response to Self-Harm.

**Figure 3:**
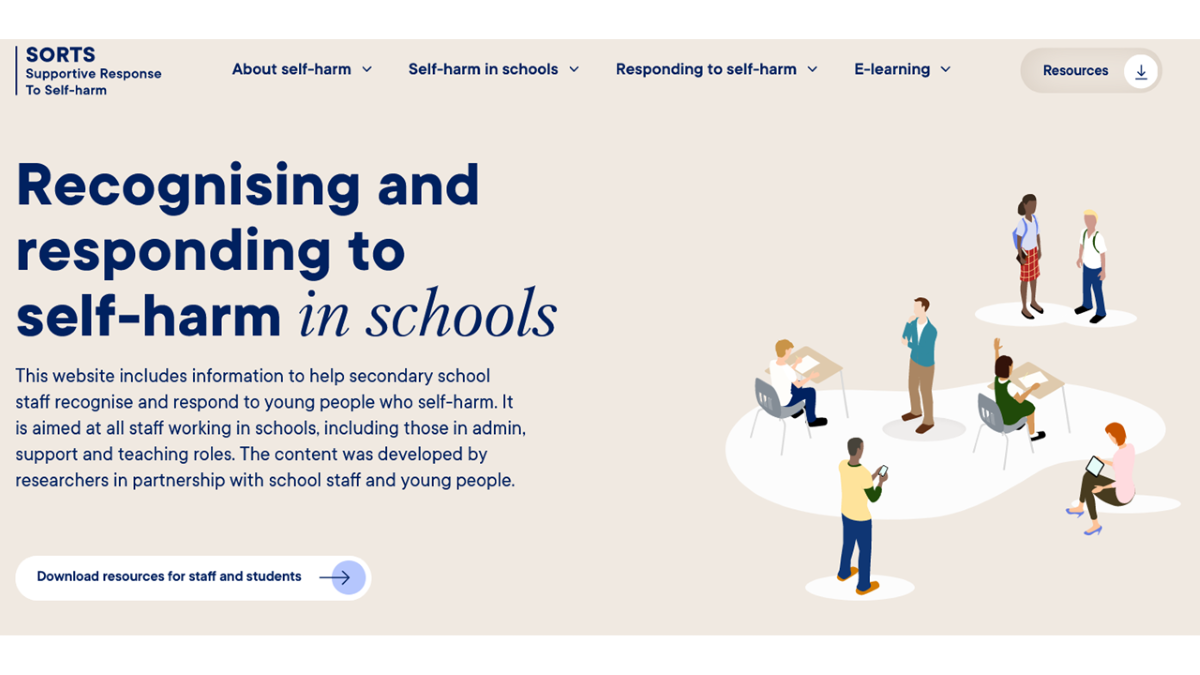
High-fidelity visual mock-up of the landing page. SORTS: Supportive Response to Self-Harm.

**Figure 4:**
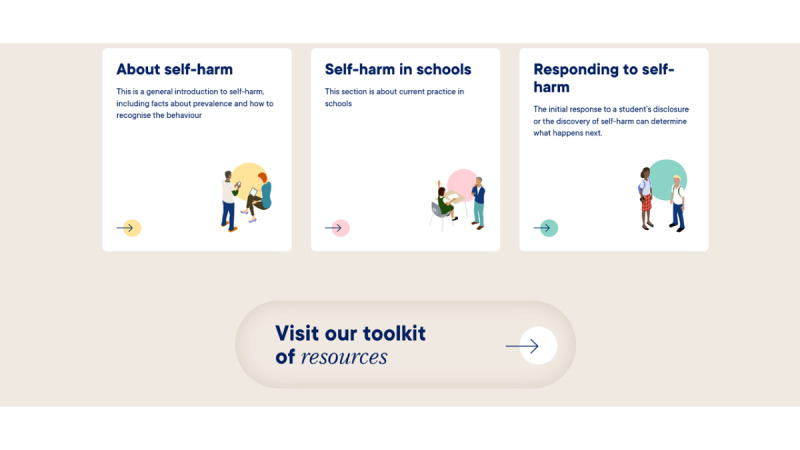
Example of proposed layout for the main sections.

Following this, participants were shown a low-fidelity mock-up of the training content; this was a hard copy of all the training modules and helped participants see the training in its entirety and how sections were connected. We explained that a user could progress through the training in a chronological order or access the individual modules. A wireframe was used to demonstrate the prototype functionality and different options for including visual and audio content on the website (eg, audio clips of young people reading quotes from the focus groups and an example video about the study that included a range of presentation styles; Figure 2). Staff brought their evaluation forms to the workshop so that they could individually provide feedback on the training content and make suggestions for other knowledge areas that they would like to be incorporated in the next iteration of the prototype. High-fidelity visual mock-ups of the proposed interface design were also presented to help generate discussions about the look and feel of the prototype (Figures 3 and 4).

### Analysis

All focus groups and workshops were recorded, transcribed professionally, and thematically analyzed using the framework method [45]. The transcripts were checked for accuracy, and all identifying information was removed before being entered into NVivo (version 12; Lumivero) for data management. An analysis plan was drawn up in more detail to guide members of the research team through the analysis. It broadly followed the following stages: transcription; familiarization with the interview; coding; and development of a working analytical framework, application of the framework, charting of data into the framework matrix, and interpretation of the data. The team (AMB, PH, and JA) began by familiarizing themselves with a subset of transcripts from each participant group and using a combination of deductive and inductive coding. Team members met to discuss how the codes could be grouped into categories, and these formed 2 initial working analytical frameworks (one for student data and one for school staff data). The working analytical frameworks were entered into NVivo and applied to all transcripts. Once all the transcripts had been coded, Microsoft Excel (Microsoft Corp) framework matrices were generated, which enabled the data to be charted by PH and AMB. This involved creating detailed summaries in each cell of the matrix while retaining key verbatim quotes. Team members met regularly to discuss potential themes and interpret the data.

## Results

### Participants in Focus Groups and Workshops

A total of 11 young people participated in the focus groups; they were aged 14-21 (mean 16.5; SD 2.38) years. All participants lived in the South East England and East of England regions and were of White ethnicity (Table 1). In total, 82% (9/11) of the young people said that they knew someone who had experienced self-harm, and 18% (2/11) reported that they had self-harmed personally.

A total of 16 school staff members (n=11, 69% female and n=5, 31% male) were recruited from 2 schools in the East of England for the workshops. The staff worked in a range of roles, and their experience ranged from 2 months to 23 years (mean 8.7 years; Table 2).

**Table 1:** Characteristics of young people in the focus groups (n=11).

Characteristics				Participants, n (%)
**Focus group 1** **(n=5; web-based setting)**				
	**Education or occupation status**			
		Secondary school	3 (60)	
		University	1 (20)	
		Full-time employment	1 (20)	
	**Sex**			
		Female	4 (80)	
		Male	1 (20)	
	**Sexual orientation**			
		Bisexual	2 (40)	
		Heterosexual	3 (60)	
**Focus group 2 (n=6; in-person secondary school setting)**				
	**Education or occupation status**			
		Secondary school	6 (100)	
	**Sex**			
		Female	3 (50)	
		Male	3 (50)	
	**Sexual orientation**			
		Bisexual	1 (17)	
		Heterosexual	2 (33)	
		Homosexual	1 (17)	
		Prefer not to say	2 (33)	

**Table 2:** Roles of school staff in co-design workshops (n=16).

Role			Participants, n (%)
**Workshop 1 (n=6)**			
	Student support assistant	3 (50)	
	SENCo^a^	1 (17)	
	Assistant head teacher (SLT^b^)	1 (17)	
	Senior tutor	1 (17)	
**Workshop 2 (n=5)**			
	Well-being lead and school counselor	1 (20)	
	Teacher	2 (40)	
	Student services manager	1 (20)	
	Mental health lead and head of year (SLT)	1 (20)	
**Workshop 3 (n=5)**			
	Teacher and head of history (SLT)	1 (20)	
	Assistant head teacher (SLT)	1 (20)	
	Teacher and behavioral support	1 (20)	
	Well-being support assistant	1 (20)	
	Student support assistant	1 (20)	

^a^SENCo: Special Educational Needs Coordinator.

^b^SLT: senior leadership team.

### Summary of Findings to Inform the SORTS Prototype

#### Overview

The analysis generated four main themes from the initial focus groups and later workshops: (1) need for a training program; (2) acceptability, practicality, and implementation; (3) design, content, and navigation; and (4) adaptations and improvements. Young people’s views were combined with staff’s views and are presented in themes 1 and 4. Staff are the end users for the training program; therefore, only staff views are presented in themes 2 and 3, which relate to the design of the training. The key themes and subthemes are presented in Textbox 1, and illustrative quotes are provided within the text.

**Themes and subthemes from the focus groups and workshops.**

**Need for a training program**

- Schools support students and parents.
- School staff need skills and knowledge to respond.
- Lack of universal training for school staff about self-harm.

**Acceptability, practicality, and implementation**

- Training content and design is engaging.
- Training content and resources were valued.
- Flexibility of training is convenient.

**Design, content, and navigation**

- Preference for simple and neutral user interface (UI) design.
- Animated artwork is preferred.
- Navigation was clear.
- Chunking of information facilitates learning.

**Adaptations and improvements**

- Information and resources are needed for parents.
- Scenario-based training is needed.
- Stand-alone e-learning modules are needed.
- Training needs to take a whole-school approach to self-harm.

#### Theme 1: Need for a Training Program

All focus group and workshop participants discussed the need for school staff to be trained on self-harm, particularly given that self-harm prevalence appeared to be increasing in their schools and within the context of long waiting times for child and adolescent mental health services. Staff described how schools are becoming a “one-stop-shop” for advice because parents are seeking information and guidance from their school before approaching health professionals:

...we’ve got young people with severe mental health challenges and they’re put in the system, and we are still there the next day, and the day after, and nothing else is happening.

Assistant head teacher; workshop 1

Concerns were raised about the potential harms of a staff member responding negatively to a student disclosure or not responding at all. Both young people and staff thought that a negative response could escalate the situation, lead to student disengagement, and prevent timely intervention and support. Some staff members admitted that they lacked confidence and knowledge about self-harm and were worried about saying or doing the wrong thing:

I’ve not personally experienced a situation as of yet—touch wood, hopefully not—but, honestly, I don’t feel like I’d be prepared to offer the right and appropriate advice if it was only me that they confided in.

Teacher; workshop 1

I’ve not got the training or the expertise. I have the confidence to talk about these things, and obviously some students find me approachable enough to tell me these things, but I don’t have the training or the expertise.

Teacher; workshop 2

Young people emphasized the importance of staff using appropriate language and being aware of the stigma associated with self-harm. They commented that “tip-toeing” around the subject only feeds the shame that young people might be feeling:

Yeah, I mean, I just think there’s a lot of shame associated with it, so avoiding language that makes people feel embarrassed or judged. I mean, I know that sounds obvious, but just approach it to be kind and, yeah...

Young person; focus group 1

Most school staff members reported that they had not received any training about self-harm and felt that this was a gap that needed to be addressed:

I can’t remember in my now 13 and a half years of teaching that I’ve had any direct self-harm training and that’s been in mainstream primary, secondary and private special education...I’d say it’s definitely a gap in my sort of professional development as a teacher, it hasn’t really been touched.

Special Educational Needs Coordinator (SENCo); workshop 1

Pastoral and safeguarding staff said that specific training about self-harm was not readily available and the topic was usually addressed as a subtopic in broader training programs about mental health or safeguarding. There was a lack of training for teachers and administrative staff:

I don’t think, not just about teaching it to the students either, because I can honestly say, I think, before you did your piece in staff briefing, in 23 years I don’t think we’ve ever had any kind of training on self-harm other than a side note to anxiety and depression in young people.

Teacher; workshop 2

A need was identified for having reliable, trustworthy, and accessible information tailored for schools:

Thinking about it with my kind of wellbeing lead hat on, I go on so many of these websites to find little reliable snippets of information to then relay back to staff, and I always want it to be easy to find and I want it to be reliable.

Mental health lead; workshop 2

Basic training for all staff should include examples of phrases to communicate well with students in the event of a disclosure. Young people discussed how students are most likely to confide or seek help from a member of staff whom they trust and feel connected to, and they said that it was important that the student feels supported in those first conversations:

I think just some basic knowledge and training of what to do if a student does approach you. It doesn’t have to be full-on, “This is what you do.” Just some basic, maybe, words to use if someone comes up to you or just the way you should speak about it.

Young person; focus group 1

If we want help with self-harm, it’s also important to consider what’s causing the self-harm and whilst it’s good to have generalised information on the causes, and such, I think it’s important that the teacher knows that each case is unique and different.

Young person; focus group 2

Although young people thought that the training should be universal to ensure a basic level of training across all staff, they noted that some staff members (eg, form tutors) may require more in-depth training as they manage students’ problems on a day-to-day basis. Physical education teachers were identified as key staff who may notice signs of a student’s self-harm and, therefore, could need an advanced level of training.

Young people thought that it was important to have clear school guidelines for staff about responding to self-harm disclosures as this would reduce students’ concerns about speaking to staff and seeking help:

The fear of the unknown is so much bigger than what’s actually going to happen, I think.

Young person; focus group 2

#### Theme 2: Acceptability, Practicality, and Implementation

In general, school staff thought that the web-based training was acceptable and practical. There was strong support from the senior staff who are responsible for student mental health policies and mental health support provision. They felt that the training aligned with the values of staff and would fit within the ethos of their school, where student mental health and well-being are a priority. Furthermore, the training program would integrate seamlessly within the school and would build staff capacity to recognize and refer students who are self-harming.

Staff expressed positive views about the training content and found the presentation design engaging and useful. They particularly valued tips about how to start conversations with students and their parents:

I literally had a situation like this yesterday. Having read some of the stuff that you guys had sent, so I couldn’t have been better-equipped for that conversation with that student, but even then I was immediately wanting to say like, “Oh, my gosh, don’t do that.” In my head, that’s my reaction that I wanted to give, knowing that’s not what I needed to give.

Teacher; workshop 3

The resource toolkit was seen as a valuable part of the program. However, there were concerns about staff appropriately using the resources, and they requested that clearer instructions be included:

I think clearer flagging might be...So resources for the individual teacher, resources for whole school.

Teacher and behavioral support; workshop 3

High workload and time constraints were identified as potential barriers for training completion. However, the format and flexibility of the web-based training were generally viewed as beneficial. Staff particularly liked that the web-based training would provide a flexibility and convenience that felt achievable in a busy school schedule and this would encourage staff to complete the training:

...as you’re well aware, teachers do have quite a hectic workload and schedule already. It’s a great idea in practice, but...I can imagine, just some teachers being like, “Well, where do I fit this? When do I have time for this?”

Teacher; workshop 2

And also, it doesn’t feel like you’ve got to find loads of time does it. An hour just feels like quite a big chunk of time in a busy day or asking people to do this in their own time if it’s an interest. Like half an hour, 20 minutes, bite sized, there’s five modules but they’re 20 minutes each feels achievable.

Assistant head teacher; workshop 1

Senior staff stated that staff could complete the training as part of their *Continuing Professional Development* (CPD), for which they have protected time:

...a universal training session for all staff is achievable within like a CPD process.

SENCo; workshop 1

#### Theme 3: Design, Content, and Navigation

Overall, staff liked the look and feel of the proposed UI design, describing it as “user-friendly” and “professional.” Staff thought that the “neutral” and “calming” color palette was appropriate and, given the nature of the topic, the color red should be avoided.

Discussions revealed that several elements of the UI were confusing (eg, the download and information icons were not intuitive and did not function as expected, and some participants suggested removing them altogether).

During the demonstration of the wireframe, participants noted that content sat below the scroll line and could easily be missed, and they recommended redesigning the home page so that the content was readily visible, thus minimizing unnecessary vertical scrolling:

I personally don’t like having to scroll to look for something. So you’ve got the things at the top, which is great, but your three boxes at the bottom, I would rather have them there in front of me.

Teacher and behavioral support; workshop 3

During the workshops, staff were shown various artwork options for the UI and an example video including various visual options. They liked the bespoke illustrations on the home page and listing pages. There was a strong preference for animations over photographic visuals, with the main reason being that animations and avatars have a timeless appeal and would stay relevant for longer. Some commented that photos can quickly become outdated and might not accurately represent all demographics; thus, potentially, some people or communities may be underrepresented:

...cartoon and imagery...it’s a bit more generic. Which is good in a way because it shows that it’s like, for everyone. It’s not just for key like demographics or key people.

SENCo; workshop 1

Senior staff emphasized the importance of the program’s underlying evidence base, which would influence their decision to implement the training in their schools. They found the university and National Health Service logos reassuring regarding the fact that the training content was supported by reputable organizations. A suggestion was to hyperlink the logos to each organization’s official website:

...to have some of those linked would be useful, so if you click on them, click on the logo, they take you to a landing page on their website.

Mental health lead; workshop 2

Participants did not think that it was necessary to have multiple videos, podcasts, or audio content, and there was a strong preference for the training program to be simple in its presentation. Including the student voice in the training content was viewed as useful and impactful; however, they would prefer written quotes rather than audio files. Participants liked that the content was presented in manageable sections:

...the document was bite-size for quick reading and informative.

However, some found that the language was “too academic” and requested that it be edited to be more accessible for school staff. Clearer headings for the training sections would help users quickly locate appropriate information, and signposting should be added to the UI to show users a logical order to complete the training.

The link to resources needed to be prominently placed on the home page for staff to access these quickly and easily. Each individual resource could be downloaded, but most staff members said that they would like an option to download the whole toolkit at once. Participants recommended reorganizing the list of resources in a more meaningful way, such as listing them alphabetically or grouped by color and clearly indicating who the resources were intended for (eg, some resources were specifically for the well-being or SLT teams):

I think clearer flagging might be...so resources for the individual teacher, resources for whole school, resources for leadership.

Teacher and behavioral support; workshop 3

The navigation bar was helpful because links were clearly labeled and provided quick and easy access to different sections of the training. However, several participants expected to see drop-down menus when hovering over the top-level links. They suggested adding drop-down menus to the navigation bar as this would match convention and it would also help users understand how the whole site is structured:

I think as well drop down menus, you said what is self-harm, you’re not clicking back five times and then you get annoyed because your browser takes too long, and you click back too many times. I want to just go back to the main bit around that, [so] you’ve got them as you hover over them.

Assistant head teacher; workshop 1

Some staff members were concerned that parents and students may come across the website and suggested adding a clear statement to the landing page that the site is specifically for school staff. Although the trigger warning on the landing page was seen as important, it would only be relevant for a user’s first visit and, therefore, could be a 1-time pop-up.

#### Theme 4: Adaptations and Improvements

Staff made several suggestions for adaptations and improvements going forward (eg, including case-study scenarios; young people’s lived experience testimonies; example conversation starters; guidance on how to develop a school self-harm policy; implementation of a whole-school approach; and information for parents, siblings, and friends). Staff described how parents are usually quite shocked, upset, or angry when they discover that their child is self-harming, and staff are intermediaries providing information and support. Expanding the website to include information and resources for parents would be helpful for schools:

Some people can keep things from their parents and it can be hard for them to accept that their child is self-harming for quite a lot of people. So I think having a lot of information for parents is just so helpful because they can actually know what could be going on.

Young person; focus group 2

Staff held the view that case-study scenarios would facilitate engagement and increase the likelihood of staff using the skills in their everyday practice. Furthermore, content presented in an e-learning module format would increase the flexibility of the training for different staff roles (eg, an induction e-learning module for new staff and a longer, more advanced or intensive e-learning module for staff in key roles such as form tutors or those with mental health responsibilities). On completion, staff would receive a certificate, and the SLT would be able to monitor training completions. The existing SORTS website would act as a useful handbook (manual) that staff could refer to:

It’s kind of like the handbook that goes with it, isn’t it? We do a lot of mental health training; they give you the physical handbook when you finish the course and it’s like the handbook’s the thing that I keep flicking back through to make sure I’m doing the right thing.

Mental health lead; workshop 2

Senior staff discussed how the training could contribute to creating a “whole school approach to self-harm,” whereby all staff, regardless of role, would be given a basic level of training to prepare them in how to react, respond, and escalate up to relevant staff. In addition to the SORTS training, schools could include psychoeducation for students about self-harm in the curriculum and develop a school self-harm policy, thereby creating a supportive environment for students to come forward for help. Young people also wanted self-harm to be included in the curriculum and for schools to implement a policy so that students have “transparency about what to expect” if they approach a staff member for help, including if a student discloses that a friend is self-harming. There was a wide acknowledgment that managing disclosures of self-harm can take its toll on staff and that there needs to be a strong support network to protect staff mental health.

### Final Prototype Specification (Develop)

The qualitative findings from the staff workshops informed the design of the final prototype specification. On the basis of participant feedback, the training content was edited to be more concise, and the language was made more accessible. The training content and resource toolkit were finalized (Textboxes 2 and 3).

**Figure 5:**
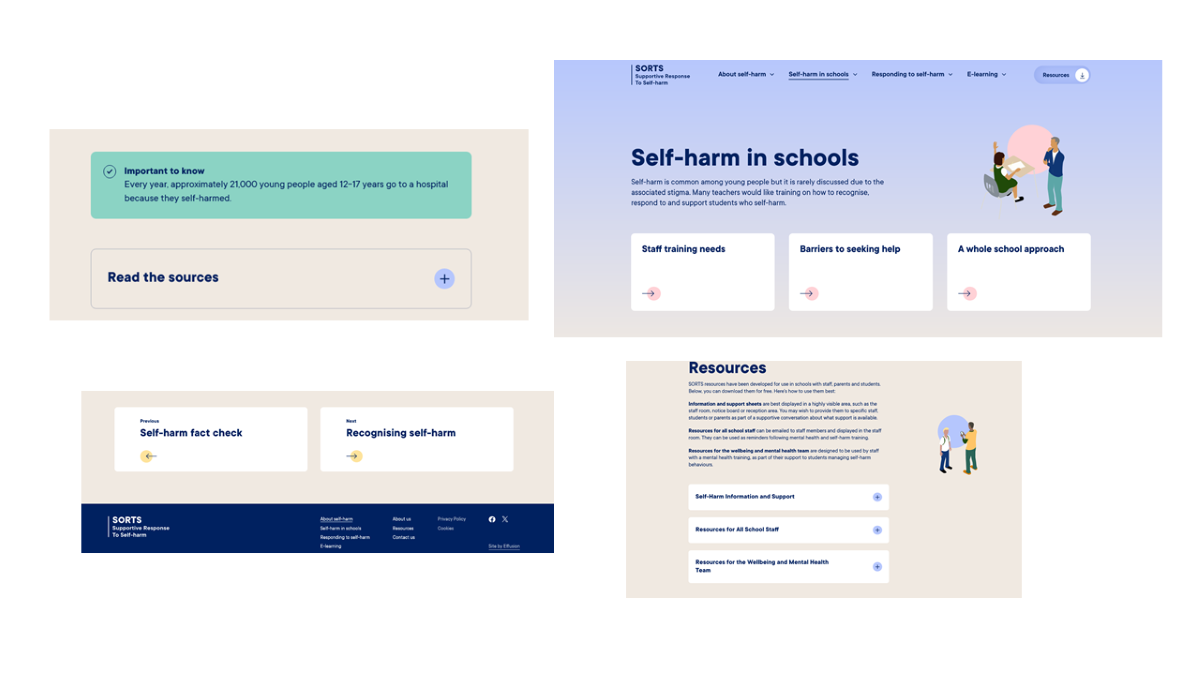
New features for final design (information modules, drop-down menus, quick-access navigation, and expandable accordion for resources). SORTS: Supportive Response to Self-Harm.

The research and technical teams worked closely to implement the changes to the website design, and a number of UI features were used to improve the content structure and presentation across both desktop and mobile platforms. Some graphical elements were removed from the UI because they did not match participants’ expectations, and new elements were added to aid navigation (eg, drop-down menus, “call to action” features, and navigation links to show a suggested direction of training modules). In response to feedback, we minimized the scroll length by introducing a carousel feature for project logos, linked the logos to organizational websites, and introduced a “download all” function for the school resource toolkit. Background and accent colors were used to separate the training topics and design elements incorporated to enhance the UI (eg, pull-out quote; 4 special information modules [*Facts*, *Warnings*, *Recommendation*, and *Did you know*]; expandable accordion for *Resources*, *Download*, and *Sources*; ways to share the page; and quick access to other pages using “Previous or Next” modules; Figure 5). Participants suggested that stand-alone e-learning modules would complement the existing training content, and these will be developed in the next iteration of intervention development.

**Supportive Response to Self-Harm training content.**

**About self-harm**

- What is self-harm?
- Why do young people self-harm?
- How common is self-harm?
- Self-harm fact check
- Self-harm risk factors
- Recognizing self-harm
- Impacts of self-harm

**Self-harm in schools**

- Staff training needs
- Barriers to seeking help
- Whole-school approach to self-harm

**Responding to self-harm**

- Initial response to self-harm
- Supportive conversations
- Long-term support for students
- Dealing with a disclosure made by friends and family
- School response flowchart
- Create your school’s self-harm guidelines

**Resource toolkit.**

**Self-harm information and support**

- Information sheet for parents
- Information sheet for students
- Support organizations for parents
- Support organizations for school staff
- Support organizations for young people

**Resources for all school staff (a whole-school approach)**

- Infographic: Cycle of self-harm
- Dos and don’ts of self-harm
- The signs of self-harm
- Responding to self-harm flowchart
- Risk factors

**Resources for the well-being or mental health team**

- Coping with big emotions
- My safety net activity
- My support plan
- Incident report form
- Follow-up letter to parents
- Guidance for developing a school self-harm policy

## Discussion

### Summary of Findings Within the Context of Wider Literature

This paper describes the collaborative design and development of the SORTS training intervention, which aims to increase school staff’s knowledge, skills, and confidence in how to respond to self-harm. We anticipate that by equipping school staff with the skills and strategies to identify and respond to self-harm, this will encourage students to seek help, prompting staff to initiate linking students with appropriate resources and support. Although the SORTS program has been developed for staff in secondary schools, it also has the potential to be integrated into other educational settings, including youth organizations.

The SORTS training program fits with the UK policy drive for schools to have responsibility for identifying students with mental health difficulties and facilitate referrals to appropriate services [10]. School staff are often the first professionals to notice whether a student is self-harming [11] and the first professionals that parents contact about their child’s well-being [46,47]. Therefore, staff are well placed to intervene and prevent escalation [16]. The review that informed this study found very few training programs and support tools for school staff to address self-harm [13]. The review’s findings showed high acceptability of the interventions and tools, improvements related to knowledge about self-harm and how to respond, confidence in responding to disclosures, and change in response to young people who disclose self-harm. However, the training interventions were poorly described, lacked follow-up data, and had limited applicability to the UK context.

The early prototype development described in this paper involved a review of the evidence base and collaborative work with different stakeholder groups, including young people, school staff, and experts. This is an ongoing iterative process, and work will continue with stakeholders in the next stage of intervention development and evaluation [40,48]. This is in line with the Medical Research Council guidance for complex interventions, which specifies that “appropriate users” should be involved at all stages of development [39]. Our framework of coproduction demonstrates how we drew upon the expertise of stakeholders in the development process to address their requirements and maximize acceptability, feasibility, and future uptake of the intervention [49]. This is particularly important for interventions intended for complex social systems such as schools [33].

Consistent with previous research, our participants reported that there is limited training for staff about self-harm [13,16-18] and some feel ill-equipped to manage a disclosure [17]. Our participants were concerned that a poor response from a staff member could potentially escalate the situation and deter students from engaging or that staff lacking in confidence may avoid responding at all, which could delay putting support in place. This resonates with previous research, which has found that staff lacking in knowledge and confidence may respond negatively [14,17,20]. Moreover, students are often reluctant to seek help because they worry about receiving a negative response [14,20,48,50]. Overall, the school staff in our study would like universal in-service training to build capacity to recognize and refer students who are self-harming and facilitate effective communication with students and parents.

Our findings also indicate that staff think that the SORTS training intervention will be acceptable and practical for schools. Staff provided feedback on the training content and UI design, which included improvements to the intervention and its implementation. In addition, they recommended developing separate scenario-based e-learning modules for future work. In line with the usability heuristics by Nielson [51], staff wanted a simple, neutral UI design and “chunking” of textual information so that it was easier for users to read and comprehend [52,53]. In response to stakeholder feedback, UI elements that did not match participants’ expectations were removed, and navigation functionality was added. We made the resources more prominent because participants highlighted that staff may need to access information quickly. Similarly, Bakker et al [54] emphasized the importance of quick access to information about crisis support in technology-based interventions.

In terms of implementation, senior staff suggested that the training could be embedded within new staff induction programs or within staff protected time for CPD. Developing scenario-based e-learning modules would provide schools with more flexibility for integrating the training into the school context. Time and cost may be potential barriers to implementing training in schools [55]; however, web-based training programs can provide flexibility and overcome these potential barriers [30].

### Whole-School Approach

**Figure 6:**
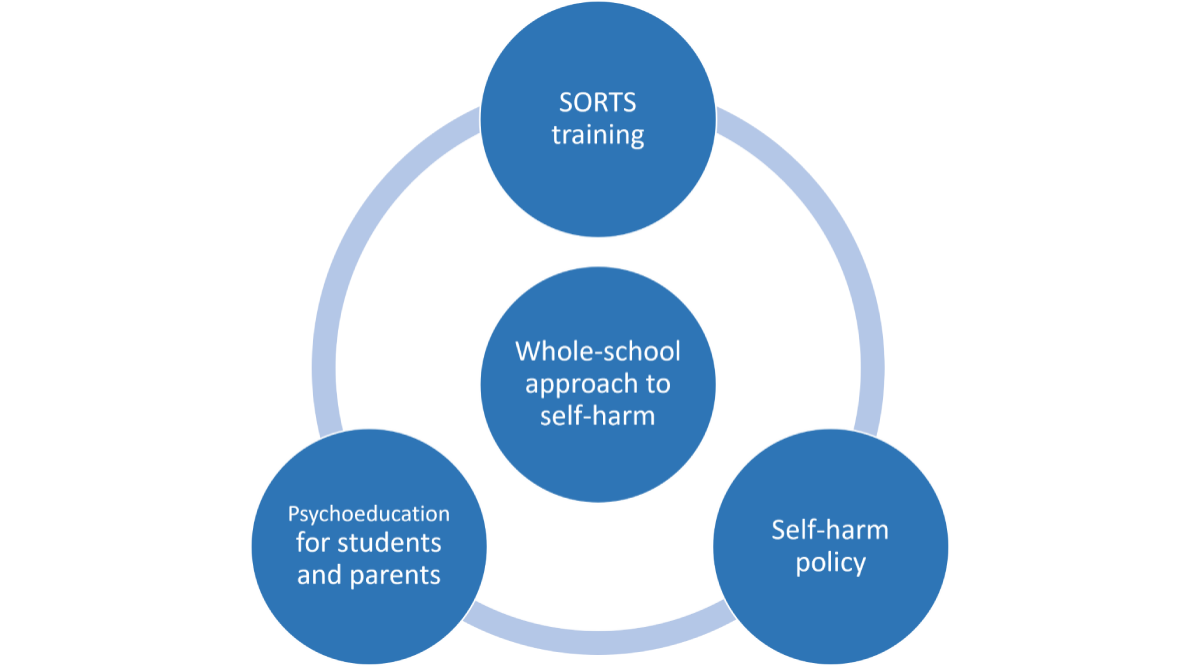
A whole-school approach to self-harm. SORTS: Supportive Response to Self-Harm.

The SORTS training program was discussed as part of a broader culture that would encourage a whole-school approach to self-harm, including psychoeducation for students and parents and developing a school policy tailored to the school context. Similarly, a recent study recommended a whole-school approach and that staff be provided with training, resources, and guidance to respond effectively to students who self-harm [56]. In our study, young people discussed how the stigma surrounding self-harm may prevent help seeking. Students are most likely to disclose to a member of staff whom they trust and feel connected to and within an environment that fosters a supportive culture for mental health. Young people felt that it was important for schools to train staff, include self-harm in the curriculum, and develop a policy so that students understand what the process will be if they disclose. On the basis of these findings, we propose that the SORTS training program be implemented as part of a whole-school approach to self-harm, which is illustrated in Figure 6.

### Strengths and Limitations

User-centered and person-based approaches offer rich information about stakeholders’ perspectives, which helps ensure that new interventions are meaningful, acceptable, and feasible. These strengths notwithstanding, there are several limitations to consider in relation to the findings. School staff were recruited from schools located in 2 towns in the East of England, and their views may differ from the views of staff in other areas of the United Kingdom and in schools situated in more rural or urban areas. Staff participants were contacted and selected by the mental health leads in each school, so it is possible that many of the staff members we spoke to had a specific interest in mental health training. A range of school staff members were included in the sample, although members of the administrative team were not represented, and their views were not captured in this study. We acknowledge that our sample of staff and young people lacked diversity in terms of ethnicity, and future work should be conducted in more ethnically diverse schools.

### Implications and Future Work

Our study highlights the importance of involving multiple stakeholders in the early stages of intervention development. In response to feedback from staff, the next stage of prototype development will involve the development of 2 scenario-based e-learning modules to be incorporated in new staff inductions and staff CPD time. The design and development of the e-learning modules will be coproduced with school staff and experts. Once intervention development is complete, we will conduct user testing of the SORTS prototype to identify and fix any usability problems. Furthermore, we will carry out a mixed methods pilot evaluation to explore acceptability, feasibility, and effectiveness of the SORTS program with specific emphasis on recruiting schools from diverse areas to explore the impact on marginalized ethnic minority groups; lesbian, gay, bisexual, transgender, and queer youth; and young people with special educational needs. Should the intervention prove effective in further evaluation, we hypothesize that school staff’s improved knowledge and confidence will lead to more appropriate responses that will encourage pupil disclosure and facilitate access to available on-site and external support. In line with a whole-school approach, the SORTS program will be expanded to provide information and resources for young people, families, and community partners.

## Appendix

Multimedia Appendix 1 Supportive Response to Self-Harm logic model.

Multimedia Appendix 2 Supportive Response to Self-Harm recruitment flyer.

Multimedia Appendix 3 Supportive Response to Self-Harm young people topic guide.

Multimedia Appendix 4 Supportive Response to Self-Harm staff workshop topic guide.

## Abbreviations

CPD: Continuing Professional Development
SENCo: Special Educational Needs Coordinator
SLT: senior leadership team
SORTS: Supportive Response to Self-Harm
UI: user interface

## Data Availability

The data sets generated and analyzed during this study are available from the corresponding author on reasonable request.
