# Supplementary figures and images for: A Web-Based Training Program for School Staff to Respond to Self-Harm: Design and Development of the Supportive Response to Self-Harm Program
Source: JMIR Form Res. 2024 Jun 4;8:e50024. doi: 10.2196/50024 (PMC11185913; doi:10.2196/50024)

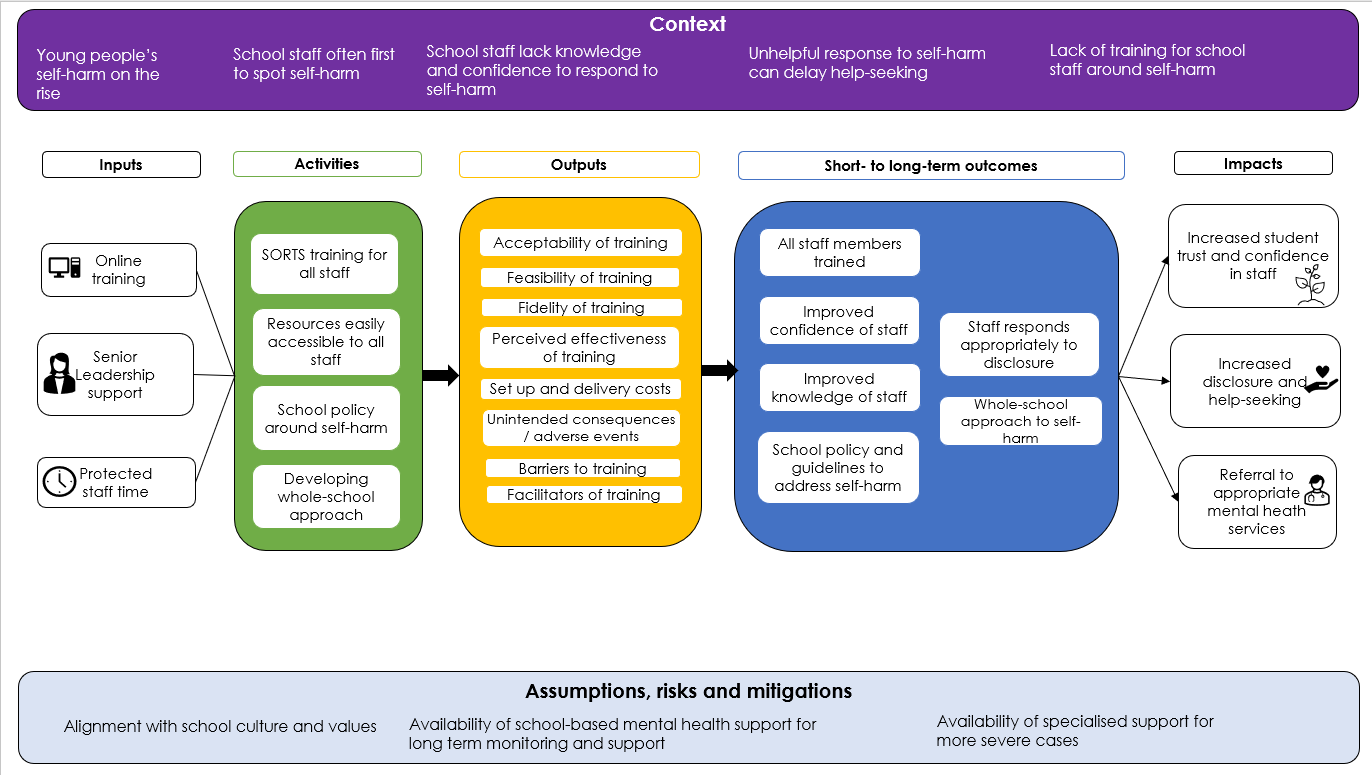

Supplement: Multimedia Appendix 1 [file formative_v8i1e50024_app1.docx]
