# Supplementary material for: A Web-Based Training Program for School Staff to Respond to Self-Harm: Design and Development of the Supportive Response to Self-Harm Program
Source: JMIR Form Res. 2024 Jun 4;8:e50024. doi: 10.2196/50024 (PMC11185913; doi:10.2196/50024)
Supplement: Multimedia Appendix 2 [file formative_v8i1e50024_app2.docx]

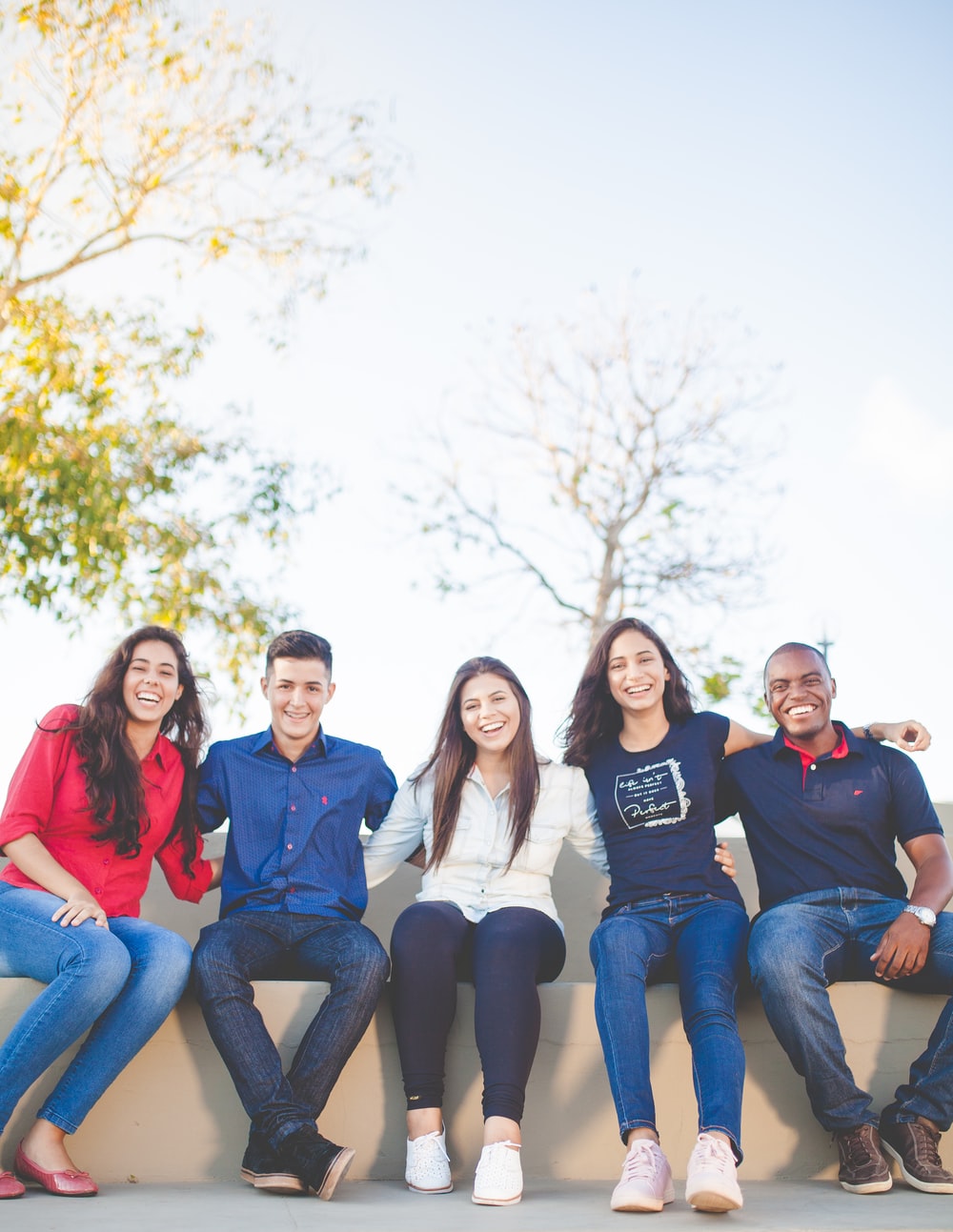


**How can schools support young people who self-harm?**

**We need to hear from young people like you!**

**Receive £20 in high street**

**vouchers as a thank you!**

We are looking for a group of enthusiastic young people to help us to understand how they think teachers should respond to young people who self-harm.


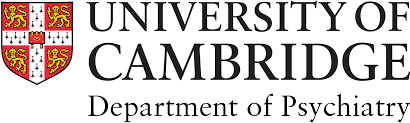


Information We would like to know how schools can support young people who self-harm. We are trying to help teachers respond better to young people who self-harm. We want to hear what young people think teachers and school staff should do if a young person is self-harming.

We are looking for young people to take part in our study who:

Taking part in the study would involve you attending a focus group or individual interview where we will discuss your thoughts about how school staff should respond to self-harm. The focus group/interview will be led by researchers from the University of Cambridge. We are interested in what you think schools could do to help support their students.

You will receive a high street shopping voucher worth £20 as a thank you for your help with the study.

If you are under 16 years of age, please ask your parent or guardian to get in touch with us for more information. If you are over 16 years of age, feel free to contact us yourself.

Contact Poppy Hall

Email: ph537@medschl.cam.ac.uk

Have not self-harmed, but are interested in how schools respond, as well as young people who have self-harmed or know someone who has.

Are aged 14-21 years old
