# Supplementary material for: A Web-Based Training Program for School Staff to Respond to Self-Harm: Design and Development of the Supportive Response to Self-Harm Program
Source: JMIR Form Res. 2024 Jun 4;8:e50024. doi: 10.2196/50024 (PMC11185913; doi:10.2196/50024)
Supplement: Multimedia Appendix 3 [file formative_v8i1e50024_app3.docx]

**Young People’s Focus Group/Interview – Topic Guide**

Structure

1 hour focus group/interview either in person or online (depending on preference).

Objective

We are exploring how young people think that school staff should address and respond to self-harm behaviour in young people.

Equipment

- Participant Information Sheet
- Consent Form
- Demographic Form

Pre-Focus Group/Interview

- Participants have read and understood the Information Sheet
- Researcher has obtained Informed Consent from young people aged 16+. For those aged 14-16 years old, assent has been obtained, as well as parental consent.

Overview:

**Introduction:**

- Research team will begin by introducing themselves and then thank all focus group/interview attendees for agreeing to take part in the study.
- Researchers will then explain how the focus group/interview will run and how long it will take.
- Researchers will remind participants that the focus group/interview will be recorded and that the research team might decide to take notes throughout.

**Consent:**

- Ask the participants individually if they have any questions and answer any that they might have.
- Remind all participants individually that they can withdraw from the focus group and from the study at any time. Remind them that the answers and information they share will be held confidentially and will not be passed on to family members or professionals (except if safeguarding issues are raised, explain clearly what this may involve, what would happen and that the participant will be involved in discussions or decisions).
- Inform all participants that we will not be asking them specifically about their own self-harm or another person’s self-harm. We will ask that participants do not share this information during the group or with the researchers. We are interested only in how young people think that school staff should respond to self-harm. Participants will be provided with a list of support organisations and websites relating to self-harm afterwards.
- Ask young people’s permission to audio record the focus group. One researcher will facilitate and a co-facilitator will take notes and bring attention to any comments in the chat (if online).
- If the young person is interested in taking part in the research but does not want to attend a focus group then they can be offered an individual interview.

**Study Rationale:**

- Remind young people of the background of the study, why the focus group/interview is happening and what will be discussed (how young people think that school staff should address and respond to self-harm in schools).

Focus Group Topic Guide

**NB:** This topic guide reflects the nature of the questions that are expected to be asked during the focus group/interview. It should be acknowledged that this is not a script, instead these questions are expected to prompt a natural discussion between researchers and young people. The conversation is expected to flow between young people in the focus groups with the encouragement of the researchers. Each question will be asked/framed in a way which is appropriately tailored to each group of young people (depending on their age range). At all times, questions and discussion points will be phrased in a sensitive manner and accordingly to the context.

**Opening: Contextual Background and Introductions (10 mins):**

- Thank young people for volunteering.
- Remind young people that they will be receiving a thank you voucher for their time.
- Ask participants to introduce themselves by sharing their first name
- Explain that during the discussions, if there is anything that they are unsure of then they can ask the researchers.

**Discussion: Experience of Self-Harm in School (45 mins):**

*Example scenario presented and YP will be given the chance to respond and feedback.*

e.g. A young person asks to speak to their music teacher privately. They inform the teacher that they have harmed themselves.

(1) What do you think the teacher should do/how should they respond? Why do you think so?

(2) What do you think the YP’s attitude/reaction toward teacher’s response? Why do you think so?

- e.g. YP notices a friend has marks on their arm and when they ask their friend about the marks their friend says that they have been self-harming. The YP is very worried about their friend and doesn’t know what to do? They start crying in a lesson and a teacher notices. The teacher asks what is wrong. The YP says that they are worried about their friend and that they think their friend is harming themselves.

(1) What do you think the teacher should do next? Why do you think so?

(2) What do you think the YP’s attitude/reaction toward teacher’s response? Why do you think so?

*If a young person wanted to receive some support for their self-harm from their school and not from home, how do you think a school could best support that young person?*

- Who, what, when, where?
- A designated person to talk to? Should all staff be approachable and know about self-harm/have the training/skills to support YP?
- A safe space/room to go to?
- Long-term support options/check-ins?

*What do you think might be potential barriers that stop young people accessing support for self-harm from their school?*

- How do YP speak to friends about SH
- Informing parents?
- Is there a clear procedure for responding to SH?
- Do you think that talking about self-harm in schools (e.g. in lessons or assemblies) will mean that more people will self-harm or start self-harming?

*What do school staff need to know about self-harm?*

- What do you think that teachers generally think about self-harm?
- What terms should schools/staff use about self-harm?
- Is there anything that staff shouldn’t say when speaking to students about self-harm (as a group or individually)?
- Do particular members of staff need to know more than others (information/training)?
- Do you think school staff need training about how to support young people who self-harm or who are affected by self-harm?
- How should a conversation about self-harm go? How would YP feel best supported?

**Closing: Sum up (5 mins):**

- Thank all participants for their time and contributions.
- Ask if there’s anything they would like to add that hasn’t been discussed.
- Inform the participants that they will receive their thank you vouchers after the focus group has ended. Ask if they would like to receive a summary report of the key findings.
- Ask if participants have any questions.
