# Supplementary material for: A Web-Based Training Program for School Staff to Respond to Self-Harm: Design and Development of the Supportive Response to Self-Harm Program
Source: JMIR Form Res. 2024 Jun 4;8:e50024. doi: 10.2196/50024 (PMC11185913; doi:10.2196/50024)
Supplement: Multimedia Appendix 4 [file formative_v8i1e50024_app4.docx]

**Topic Guide – Staff Workshops**

**Structure**

2 hour co-production workshop either in person or online (depending on preference).

**Objective**

We are exploring school staff’s feedback on the SORTS training programme prototype. We will be discussing how school staff should address and respond to self-harm behaviour in young people and what kind of training would be helpful to them.

**Equipment**

- Participant Information Sheet
- Consent Form
- Demographic Form

**Pre-Co-production workshop**

- Participants have read and understood the Information Sheet
- Researcher has obtained Informed Consent from all participants.
- Prior to the workshop, participants will be asked to explore the SORTS website and will be asked to complete some tasks to ensure they visit different pages and experience the different elements. For example, they will be asked to complete one of the modules, watch a lived experience video and complete a quiz. We will ask participants to keep note of usability issues they encounter.

**Overview:**

**Introduction:**

- Research team will begin by introducing themselves and then thank all co-production workshop attendees for agreeing to take part in the study.
- Researchers will then explain how the workshop will run and how long it will take.
- Researchers will remind participants that the workshop will be recorded and that the research team might decide to take notes throughout.

**Consent:**

- Ask the participants individually if they have any questions and answer any that they might have.
- Remind all participants individually that they can withdraw from the workshop and from the study at any time. Remind them that the answers and information they share will be held confidentially and will not be passed on (except if safeguarding issues are raised, explain clearly what this may involve, what would happen and that the participant will be involved in discussions or decisions).
- Inform all participants that we will not be asking them about individual student’s self-harm. We will ask that participants do not share this information during the group or with the researchers.
- Ask young participant’s permission to audio record the workshop. One researcher will facilitate and a co-facilitator will take notes and bring attention to any comments in the chat (if online).

**Study Rationale:**

- Remind participants of the background of the study, why the workshop is happening and what will be discussed (the online training programme for school staff – supportive response to young people that self-harm).

**Workshop Topic Guide**

**NB:** This topic guide reflects the nature of the questions that are expected to be asked during the workshop. It should be acknowledged that this is not a script, instead these questions are expected to prompt a natural discussion between researchers and young people. The conversation is expected to flow between young people in the workshop with the encouragement of the researchers. At all times, questions and discussion points will be phrased in a sensitive manner and accordingly to the context.

**Opening: Contextual Background and Introductions (10 mins):**

- Thank participants for volunteering.
- Remind participants that they will be receiving a thank you voucher for their time.
- Ask participants to introduce themselves by sharing their first name and their job role.
- Explain that during the discussions and feedback, if there is anything that they are unsure of then they can ask the researchers.

**Your views about self-harm in schools and supporting young people (slide 5)**

- How do you think self harm should be addressed in schools? (taught as part of the curriculum? PSHE, assemblies)
- How comfortable/confident are you having conversations with YP about self-harm? (say, do) who to speak in the school)
- What concerns if any, do you have about talking to a pupil who is self-harming? Do you think some staff will find this difficult to deal with?
- Do you think it’s a good idea to train every member of staff in how to respond? (e.g. admin, teaching and supportive)

**Front page (slide 6)**

- What are your first thoughts and impressions about the design?
  - colour scheme, artwork, layout
- What do you like about it?
- What changes would you suggest?

**Navigating the website – Wireframe (slide 7)**

- Do you think the structure looks easy to navigate?
- Is there anything that you think would make it easier to move around?
- It highlights as you go over the box – do you like that?
- Are there any things on other websites that you really don’t like and we should avoid including?

**Website content feedback (slide 9)**

- Each participant will have read through a section and can feedback and discuss with the group
- Is the content informative?
- Is it helpful/interesting?
- What would you like to see more of/change?
- Any suggestions?

**Video (slide 10)**

- What do you think of having a video?
- The video contains different styles – which style do you prefer?
- How many videos should we have?

**Audio Recordings (slide 11)**

- What are your first thoughts and impressions?
- Are these impactful?
- How many should we have?
- How often should they appear on the website? Where?

**Final thoughts**

- Amount of content realistic for someone to get through?
- Are there any practical things that will stop people using this training?
- Kept informed if new material is added?
- Do you have any final comments or questions?

**Closing: Sum up (5-10 mins):**

- Thank all participants for their time and contributions.
- Ask if there’s anything they would like to add that hasn’t been discussed.
- Inform the participants that they will receive their thank you vouchers after the workshop has ended. Ask if they would like to receive a summary report of the key findings.
- Ask if participants have any questions.
